# Metal implants on abdominal CT: does split-filter dual-energy CT provide additional value over iterative metal artifact reduction?

**DOI:** 10.1007/s00261-022-03682-3

**Published:** 2022-09-30

**Authors:** Hildegard M. Wichtmann, Kai R. Laukamp, Sebastian Manneck, Konrad Appelt, Bram Stieltjes, Daniel T. Boll, Matthias R. Benz, Markus M. Obmann

**Affiliations:** 1 Clinic of Radiology and Nuclear Medicine, University Hospital Basel, University of Basel, Petersgraben 4, CH-4031 Basel, Switzerland; 2 Department of Diagnostic and Interventional Radiology, University Hospital Cologne, Cologne, Germany; 3 Department of Molecular and Medical Pharmacology, University of California Los Angeles, Los Angeles, CA USA

**Keywords:** Dual-energy x-ray computed tomography, Arthroplasty, Artifacts, Hip, Spine

## Abstract

**Purpose:**

To assess image quality and metal artifact reduction in split-filter dual-energy CT (sfDECT) of the abdomen with hip or spinal implants using virtual monoenergetic images (VMI) and iterative metal artifact reduction algorithm (iMAR).

**Methods:**

102 portal-venous abdominal sfDECTs of patients with hip (*n* = 71) or spinal implants (*n* = 31) were included in this study. Images were reconstructed as 120kVp-equivalent images (Mixed) and VMI (40–190 keV), with and without iMAR. Quantitative artifact and image noise was measured using 12 different ROIs. Subjective image quality was rated by two readers using a five-point Likert-scale in six categories, including overall image quality and vascular contrast.

**Results:**

Lowest quantitative artifact in both hip and spinal implants was measured in VMI_190keV-iMAR_. However, it was not significantly lower than in Mixed_iMAR_ (for all ROIs, *p* = 1.00), which were rated best for overall image quality (hip: 1.00 [IQR: 1.00–2.00], spine: 3.00 [IQR:2.00–3.00]). VMI_50keV-iMAR_ was rated best for vascular contrast (hip: 1.00 [IQR: 1.00–2.00], spine: 2.00 [IQR: 1.00–2.00]), which was significantly better than Mixed (both, *p* < 0.001). VMI_50keV-iMAR_ provided superior overall image quality compared to Mixed for hip (1.00 vs 2.00, *p* < 0.001) and similar diagnostic image quality for spinal implants (2.00 vs 2.00, *p* = 0.51).

**Conclusion:**

For abdominal sfDECT with hip or spinal implants Mixed_iMAR_ images should be used. High keV VMI do not further improve image quality. IMAR allows the use of low keV images (VMI_50keV_) to improve vascular contrast, compared to Mixed images.

**Graphical abstract:**

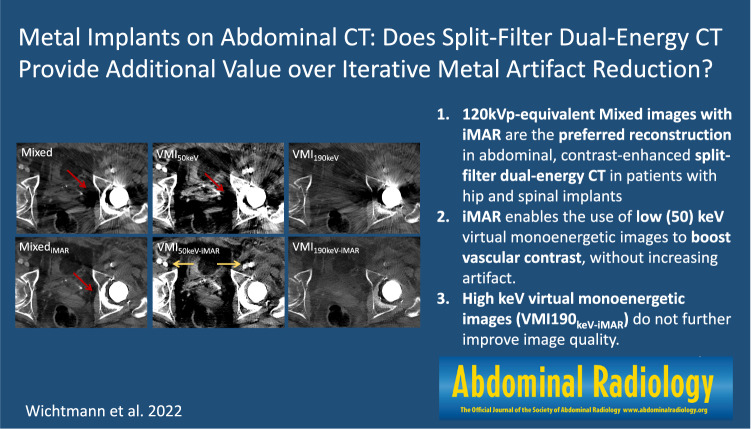

**Supplementary Information:**

The online version contains supplementary material available at 10.1007/s00261-022-03682-3.

## Introduction

Both hip fractures and vertebral fractures are common in the aging patient population. Therefore, metal hip replacements and spinal stabilizations are often encountered in abdominal imaging. Metal generates typical high- and low-density streak artifacts on CT [1]. Dedicated iterative metal artifact reduction algorithms (iMAR) are available to reduce metal artifact for a variety of implants [2–8].

Another approach to reduce metal artifacts is dual-energy CT (DECT) derived virtual monoenergetic images (VMIs) [9–11]. They simulate an acquisition with a monoenergetic beam. Higher energies than that of the polyenergetic beam can be simulated, without actually increasing the tube voltage of the acquisition [12]. Currently there are different hardware solutions available to acquire DECT images [13]. Multiple studies have shown the feasibility of metal artifact reduction with high keV VMIs for most DECT scanners [14–23]. The combination of iMAR and VMIs on established DECT platforms has shown different image reconstructions to be most favorable [8, 23–29]. Nevertheless, there is a paucity of studies on the metal artifact reduction capabilities of the split-filter DECT platform (Twin Beam DECT, sfDECT). SfDECT uses a filter made of equal parts of gold and tin in front of the tube output, splitting the beam in a high- and low energy part [30]. The tin part of the filter shifts the x-ray spectrum toward higher energies, leading to a higher dose efficiency and possibly aiding imaging of metal implants [31]. Because of the limited spectral separation of the sfDECT [32], it remains unclear if high keV VMI will be of added value for spinal and hip implants if iMAR is used.

Previous studies of DECT have focused on high energy VMI to reduce metal artifact, since low energy VMI increase artifact. However, in abdominal imaging mostly low energy VMIs are employed to investigate clinical questions: Improve vascular contrast and create arterial phase-like images from venous phase images [33], increase conspicuity of pancreatic or hepatic lesions [34], bowel wall ischemia [35, 36], or sensitivity in CT colonography [37]. Using iMAR may be a way to enable the use of low keV VMI with metal implants.

The aim of this study was to investigate if iMAR, VMIs or their combination are able to improve objective and subjective image quality over 120kVp-equivalent images in split-filter abdominal DECT with hip replacements or spinal hardware.

## Materials and methods

### Study population

**Fig. 1:**
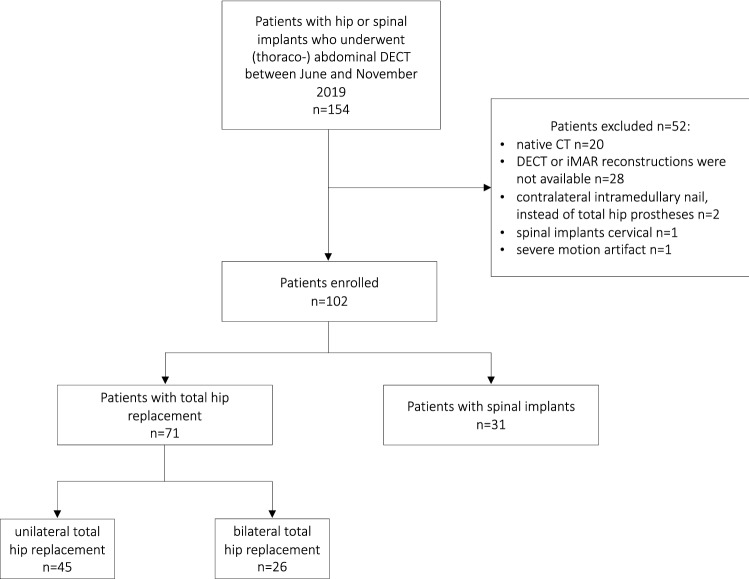
Study population: 154 consecutive patients with either hip or spinal implants, who underwent abdominal sfDECT between June and November 2019, were initially included. Of these 154 patients, 52 patients were excluded from analysis. The final study population for analysis comprised a total of 102 patients

This study was approved by our institutional review board and the need for informed consent was waived. Between June 2019 and October 2019, 154 consecutive adult patients with either hip or spinal metal implants, who underwent portal venous phase (thoraco-)abdominal DECT for other clinical reasons were initially included in this retrospective study (Fig. 1).

### DECT Imaging and postprocessing

All patients underwent portal venous phase (thoraco-)abdominal DECT and received a non-ionic contrast medium (Ultravist® 370 mg I/ml, Bayer HealthCare Pharmaceuticals, Berlin, Germany or Iopamiro® 370 mg I/ml, Bracco Suisse S.A., Manno, Switzerland) at a volume between 60 and 115 ml, adjusted to patient weight, with a flow of 1.5–3 ml/s, intravenously. All exams were performed on sfDECT scanners (SOMATOM Definition Edge (*n* = 31), or SOMATOM Definition AS + (*n* = 71), Siemens Healthineers, Erlangen, Germany). The following DECT acquisition parameters were applied: tube voltage 120 kVp, split-beam tube filtration with gold and tin, 420 average reference mAs, 64 × 0.6 mm collimation, 0.33 s rotation time and 0.3 pitch. All acquisitions were obtained with automatic tube current modulation (CARE Dose4D; Siemens Healthineers).

The dual-energy source images were reconstructed using ADMIRE 3 with a Q30f kernel at 1.5 mm slice thickness with 1 mm slice interval, with and without iMAR (hip or spine setting, depending on the implant present), as a high- and low-kVp dataset at the scanner console and automatically transferred to a post processing software (syngo.via VB30A_HF04, Siemens Healthineers). In the “CT Dual-Energy” workflow conventional- or 120kVp-equivalent images were generated with vendor recommended settings as a linear blend of the high-(20%) and low-(80%) energy dataset, further called Mixed images. Using the “Monoenergetic Plus” application with vendor recommended settings (resolution = 6, Minimum [HU] =  − 950, Maximum [HU] = 3071) VMI were reconstructed at keV levels ranging from 40 to 190 keV with an 10 keV increment. In the syngo.via software all datasets were reconstructed at 5 mm slice thickness with 2.5 mm slice interval (Table 1).

**Table 1:** Image acquisition and- reconstruction parameters

Acquisition parameter	Setting			
Tube voltage [kVp]	AuSn 120			
Reference mAs [mAs]	420			
Dose modulation	CARE DOSE 4D			
Collimation [mm]	64 × 0.6			
Pitch	0.3			
Rotation time [s]	0.33			

Reconstruction	Mixed	Mixed iMAR	VMI	VMI iMAR
Reconstruction algorithm	ADMIRE 3	ADMIRE 3	ADMIRE 3	ADMIRE 3
Kernel	Q30f	Q30f	Q30f	Q30f
Metal artifact reduction	–	iMAR	–	iMAR
Slice thickness [mm]	5	5	5	5
Slice interval [mm]	2.5	2.5	2.5	2.5

### Objective image quality measurements

**Fig. 2:**
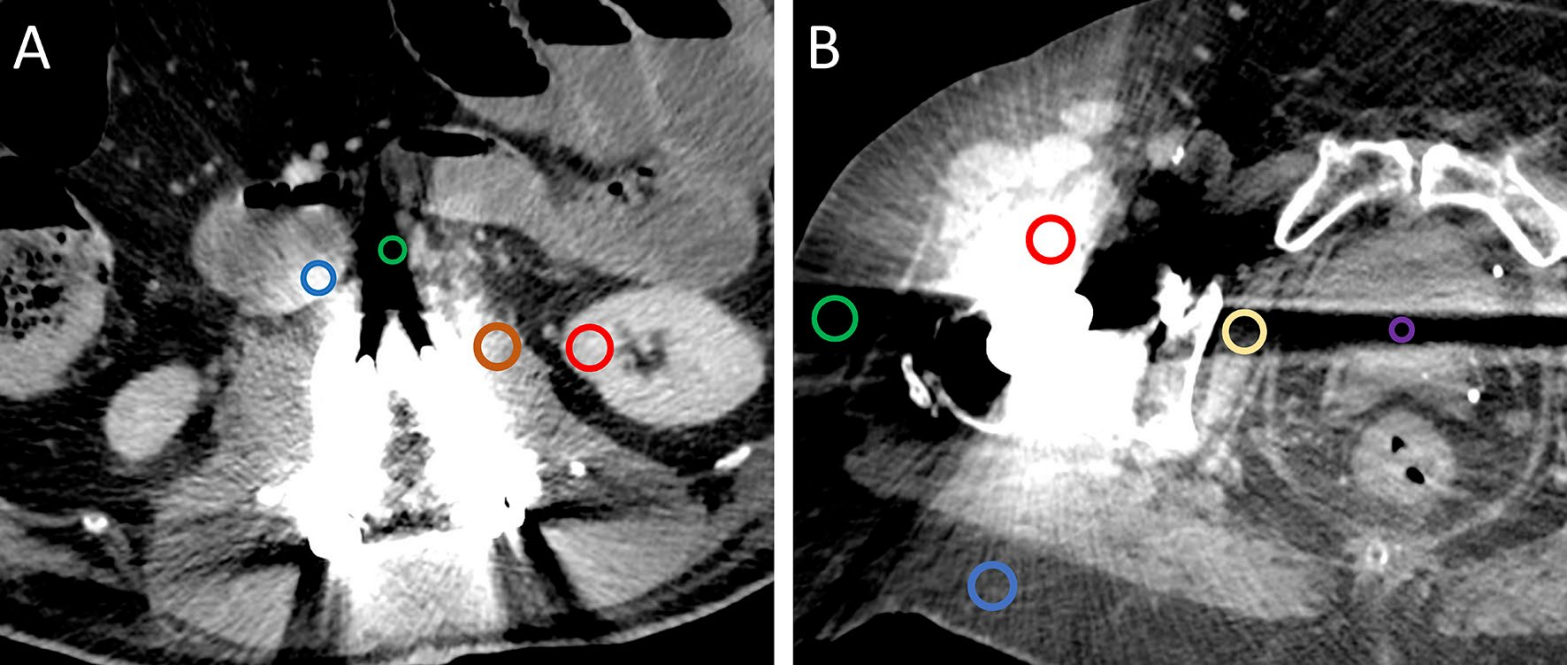
Example of ROI placements in a patient with **a** spinal implants and **b** bilateral hip prostheses. **a** Exemplary positioning of ROIs in the hyperdense artifacts in inferior vena cava (blue circle), psoas muscle (brown circle) and the slight hyperdense artifact in kidney (red circle), as well as in the hypodense artifact in the abdominal aorta (green circle). ROIs in the subcutaneous fat were placed in a different slice not displayed in the image. **b** ROIs were placed in the hypodense artifact in bladder (purple circle), as well as in by hyper- (blue circle) and hypodense (green circle) artifact impaired subcutaneous tissue. The hyperdense artifact in muscle was measured in the iliopsoas muscle (red circle), the hypodense muscle artifact (beige circle) was measured in the internal obturator muscle. The ROI to measure the hyperdense artifact in bladder was placed in a different slice not displayed in the image. All measurements were corrected by calculating the difference between artifact impaired tissue and its reference tissue without artifact (not captured in this image)

For objective image quality measurements images were transferred to a secondary imaging platform, “Nora Imaging” [38]. This enabled the placement of circular regions of interest (ROIs) in the exact same image position for all 34 different image reconstructions of each patient. ROIs were placed on axial image reconstructions of mixed images without iMAR and then automatically copied to all other reconstructions. The size of the ROIs was set to 1 cm^2^, and modified to capture only the artifact and only the specific underlying tissue. ROIs were placed in artifacts on clearly defined tissue as well as farther away reference tissue not affected by artifacts. The specific ROI positions for the evaluation of hip prostheses and spinal implants are described in Table 2 and displayed in Fig. 2.

**Table 2:** ROI positions

	Hypodense artifacts	Hyperdense artifacts	Artifact-free
Hip prosthesis	Internal obturator muscle	Iliopsoas muscle	Muscle
	Bladder	Bladder	Bladder
	Subcutaneous fat	Subcutaneous fat	Subcutaneous fat
Spinal implants	Abdominal aorta		Abdominal aorta
		Inferior vena cava	Inferior vena cava
	Subcutaneous fat	Subcutaneous fat	Subcutaneous fat
		Kidney	Kidney
		Psoas muscle	Psoas muscle

In all ROIs mean attenuation in Hounsfield Units (HU) and standard deviation values were automatically extracted. To account for the influence of monoenergetic reconstructions on measured HU values, the corrected attenuation was calculated as the difference between artifact impaired tissue and its reference tissue without artifact to only take the real artifact into account, as previously described in the literature [23]:

$$Artifact\left[ {HU} \right]_{(corrected)} = Artifact\left[ {HU} \right]_{(uncorrected)} - Underlying \, tissue\left[ {HU} \right]$$

As a surrogate for image noise standard deviation (SD) of attenuation in HU was measured in tissues affected by artifact and corrected by measurements of the same tissue in areas without artifact, as previously suggested [39]:

$$Image \, noise\left[ {HU} \right] = SD \, of \, artifact\left[ {HU} \right] - SD \, of \, same \, underlying \, tissue \, without \, artifact\left[ {HU} \right]$$

### Subjective image quality assessment

Two board-certified radiologists with 5 and 6 years of training rated subjective image quality. Readers were trained on 8 datasets from patients excluded from the analysis. Readings were performed on Mixed and VMIs at 50, 70, 110, 140, and 190 keV both with and without iMAR. Window width/level settings could be freely adjusted. Readers evaluated artifacts and vascular contrast on a five-point-Likert-scale, with a score of one representing only subtle artifact/excellent vascular contrast and five representing massive artifacts/vascular contrast similar to the use of no intravenous contrast. Depending on the evaluated tissue individual scores were further specified for the readers (Supplementary Table 1).

### Statistical analysis

To assess inter-reader agreement of subjective reading, the intraclass correlation coefficient (ICC) was calculated as a two-way random effects model as a mean of the two raters for absolute agreement, and interpreted as previously reported [40, 41]. Paired t-tests were used to compare quantitative artifact measurements of 40–190 keV vs 40–190 keV_iMAR_ of the same keV level, as well as Mixed vs Mixed_iMAR_. One-way ANOVA with Tukey honestly significant difference post-hoc test was used to compare quantitative artifact and image noise between Mixed and 40–190 keV, as well as Mixed_iMAR_ and 40–190 keV_iMAR_.

Subjective readings were compared using Kruskal–Wallis H test. Differences between individual image reconstructions were further investigated using a pairwise Mann–Whitney test with Benjamini–Hochberg adjustment to correct for multiple testing. Additionally, in the subgroup of patients, where the metal artifact was at the site of clinical question, differences in overall image quality were compared between Mixed and Mixed_iMAR_ using the pairwise Mann–Whitney test. The level of statistical significance was defined as *p* < 0.05. Statistical analysis was performed using R (Version 4.0.5) [42].

## Results

### Study population

Of the initially included 154 consecutive patients a total of 52 patients were excluded from analysis due to different reasons (Fig. 1).

Thus, the final study population for analysis comprised a total of 102 consecutive patients (female *n* = 55, male *n* = 47), with a mean age of 77 years (range 50–96 years). Clinical indications for the included scans varied: Infection (*n* = 54), oncologic (*n* = 24), intestinal obstruction (*n* = 21), trauma (*n* = 2), ischemia (*n* = 1). 71 patients had hip implants (unilateral *n* = 45, bilateral *n* = 26), 31 patients had spinal implants. 37% of patients (38/102) had a specific clinical question and/or finding in a site affected by the metal implants (32 hip implants and 6 spinal implants). The average weight and body mass index (BMI) were 72.7 ± 17.9 kg and 26.0 ± 6.3 kg/m^2^ for the hip implant group and 73.9 ± 19.5 kg and 25.5 ± 5.13 kg/m^2^ for the spinal implant group, respectively. The average radiation dose for abdominal scans was: CTDIvol 10.90 ± 3.11 mGy and DLP 579.48 ± 202.39 mGy*cm.

### Objective image quality

#### Hip implants

For images without iMAR the keV level of VMI with the lowest artifact was VMI_190keV_ for all measured ROIs (Table 3), (Supplementary Table 2a).

**Table 3:** Quantitative artifact hip implants

	Muscle hyperdense artifact	Muscle hypodense artifact	Subcutan hyperdense artifact	Subcutan hypodense artifact	Bladder hyperdense artifact	Bladder hypodense artifact
Mixed	206.51 ± 105.16	− 260.05 ± 141.4	52.74 ± 29.37	− 133.54 ± 77.54	85.16 ± 95.85	− 180.31 ± 139.37
VMI_50keV_	453.92 ± 366.29	− 526.81 ± 293.63	113.82 ± 112.48	− 321.6 ± 180.64	174.62 ± 248.51	− 412.61 ± 320.33
VMI_70keV_	271.61 ± 155.68	− 333.55 ± 176.31	67.05 ± 46.65	− 184.79 ± 100.81	108.88 ± 129.13	− 247.03 ± 194.23
VMI_110keV_	160.6 ± 105.13	− 209.74 ± 134.02	38.84 ± 26.97	− 101.81 ± 66.99	70.05 ± 82.79	− 133.82 ± 111.07
VMI_140keV_	135.67 ± 116.35	− 181.9 ± 134.28	32.39 ± 30.36	− 83.15 ± 63.94	61.2 ± 79.11	− 107.85 ± 98.4
VMI_190keV_	120.17 ± 126.32	− 164.33 ± 136.68	28.3 ± 33.79	− 71.33 ± 63.23	55.53 ± 78.65	− 91.44 ± 92.89
Mixed_iMAR_	2.59 ± 29.14	− 18.37 ± 69.7	16.45 ± 18.9	3.71 ± 18.68	4.45 ± 20.13	− 12.09 ± 16.89
VMI_50keV-iMAR_	− 7.22 ± 126.6	− 30.55 ± 212.37	31.99 ± 79.37	− 4.52 ± 60.64	15.73 ± 55.84	− 16.8 ± 50.31
VMI_70keV-iMAR_	0.69 ± 50.18	− 21.24 ± 106.46	18.71 ± 30.25	− 0.73 ± 26.33	7.33 ± 27.31	− 13.43 ± 24.01
VMI_110keV-iMAR_	4.38 ± 26.78	− 14.83 ± 50.72	10.69 ± 19.12	1.72 ± 19.51	2.3 ± 17.47	− 11.24 ± 14.75
VMI_140keV-iMAR_	5.28 ± 31.39	− 13.39 ± 41.97	8.79 ± 22.98	2.29 ± 21.63	1.16 ± 17.57	− 10.78 ± 15.04
VMI_190keV-iMAR_	5.84 ± 35.74	− 12.52 ± 38.2	7.64 ± 26.18	2.66 ± 23.49	0.44 ± 18.17	− 10.5 ± 15.74
*p*-values						
Mixed vs VMI_190keV_	1.00	0.047	0.819	0.003	1.00	0.058
Mixed vs Mixed_iMAR_	< 0.001	< 0.001	0.064	< 0.001	0.018	< 0.001
Mixed_iMAR_ vs VMI_190keV_	0.003	< 0.001	1.00	< 0.001	0.772	0.195
VMI_190keV-iMAR_ vs VMI_190keV_	0.006	< 0.001	0.972	< 0.001	0.613	0.163
Mixed_iMAR_ vs VMI_190keV-iMAR_	1.00	1.00	1.00	1.00	1.00	1.00
VMI_50keV-iMAR_ vs Mixed	< 0.001	< 0.001	0.970	< 0.001	0.127	< 0.001
VMI_50keV-iMAR_ vs VMI_190keV_	0.001	< 0.001	1.00	0.001	0.987	0.315
VMI_50keV-iMAR_ vs VMI_190keV-iMAR_	1.00	1.00	0.825	1.00	1.00	1.00

Corrected quantitative artifact as mean ± standard deviation in HU in patients with hip implants, for image reconstructions which were also rated for subjective image quality. A complete table with VMI reconstructions for all keV steps can be found the supplementary material (supplementary Table 2a). Comparisons for displayed *p*-values were made with one-way ANOVA with Tukey honestly significant difference post-hoc test

Comparing Mixed_iMAR_ to Mixed images, Mixed_iMAR_ showed decreased artifact in all ROIs. This difference reached statistical significance for all ROIs (*p* < 0.05), except for hyperdense artifact in subcutaneous fat (*p* = 0.06). Lowest artifact in VMI_iMAR_ were observed in VMI_190keV-iMAR_, albeit not being statistically significant for any of the ROIs (for all *p* > 0.05).

**Fig. 3:**
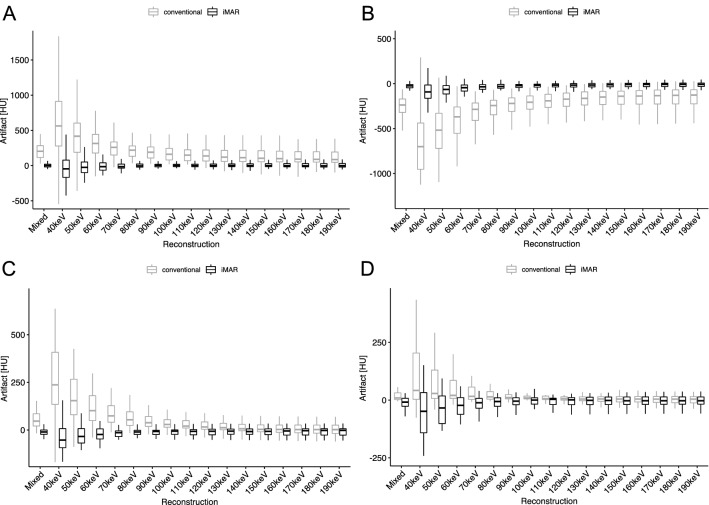
Boxplots of corrected Metal artifact for different reconstructions with and without iMAR. **a** Strongest hyperdense artifact in muscle in scans with hip prostheses. **b** Hypodense artifact in the internal obturator muscle in scans with hip implants. **c** Hyperdense artifact in the inferior vena cava in scans with spinal implants. **d** Hyperdense artifact in the kidney in scans with spinal implants

Slight overcorrection of artifacts was seen in iMAR images in hyperdense artifact in muscle (Mean corrected artifact VMI_40keV-iMAR_: − 15.26 HU) (Fig. 3a), as well as the hypodense artifact in subcutaneous tissue (Mean corrected artifact VMI_190keV-iMAR_: 2.66 HU).

VMI_50keV-iMAR_ showed less artifact in all ROIs compared to the optimal VMI images without iMAR (VMI_190keV_) (Fig. 3b). VMI_50keV-iMAR_ images showed a stronger artifact reduction than Mixed images without iMAR. Comparison of VMI_50keV-iMAR_ to VMI_190keV-iMAR_ showed no statistically significant difference in artifact reduction (*p* > 0.83 for all ROIs).

#### Spinal implants

In images without iMAR lowest artifact was observed in VMI_190keV_ (Supplementary Table 2b). However, there was no statistically significant difference compared to Mixed images (all *p* > 0.08) (Table 4).

**Table 4:** Quantitative artifact spinal implants

	Abdominal aorta hypodense artifact	IVC hyperdense artifact	Subcutan hypodense artifact	Subcutan hyperdense artifact	Kidney hyperdense artifact	Psoas muscle hyperdense artifact
Mixed	− 304.11 ± 166.93	56.14 ± 45.35	− 85.46 ± 66.91	77.64 ± 53.02	17.55 ± 31.95	77.5 ± 88.49
VMI_50keV_	− 727.41 ± 323.81	176.25 ± 157.45	− 164.14 ± 136.42	209.4 ± 152.81	60.15 ± 112.43	258.19 ± 236.38
VMI_70keV_	− 403.86 ± 201.29	85.3 ± 70.35	− 104.71 ± 79.02	108.76 ± 73.29	29.52 ± 51.24	120.74 ± 124.89
VMI_110keV_	− 204.14 ± 145.95	27.75 ± 32.99	− 67.31 ± 57.59	45.31 ± 40.08	9.24 ± 19.01	35.31 ± 62.36
VMI_140keV_	− 159.09 ± 139.1	14.48 ± 33.88	− 58.81 ± 55.3	31.12 ± 39.66	4.76 ± 16.41	15.85 ± 51.24
VMI_190keV_	− 130.62 ± 136.22	6.13 ± 37	− 53.47 ± 54.5	22.1 ± 41.25	1.94 ± 17.1	3.57 ± 45.68
Mixed_iMAR_	− 54.55 ± 115.44	− 10.44 ± 32.39	− 8.63 ± 40.39	42.01 ± 38.06	− 13.53 ± 30.2	15.55 ± 51.14
VMI_50keV-iMAR_	− 70.28 ± 252.32	− 21.98 ± 119.93	2.69 ± 132.04	92.8 ± 115.88	− 34.47 ± 72.84	43.06 ± 145
VMI_70keV-iMAR_	− 57 ± 155.01	− 12.55 ± 50.95	− 6.34 ± 60.59	54.13 ± 53.07	− 16.43 ± 37.48	22.1 ± 72.92
VMI_110keV-iMAR_	− 47.36 ± 82.83	− 7.45 ± 21.77	− 11.42 ± 24.65	28.63 ± 33.27	− 6.26 ± 29.69	9.51 ± 38.32
VMI_140keV-iMAR_	− 44.81 ± 70.37	− 6.43 ± 23.44	− 12.48 ± 23.49	22.9 ± 35.1	− 3.9 ± 30.43	6.62 ± 35.31
VMI_190keV-iMAR_	− 43.1 ± 63.88	− 5.75 ± 26.55	− 13.14 ± 25.34	19.29 ± 37.5	− 2.43 ± 31.36	4.76 ± 34.93
*p*-values						
Mixed vs VMI_190keV_	0.083	0.766	1.00	0.609	1.00	0.658
Mixed vs Mixed_iMAR_	< 0.001	0.153	0.039	0.998	0.959	0.928
Mixed_iMAR_ vs VMI_190keV_	1.00	1.00	0.936	1.00	1.00	1.00
VMI_190keV-iMAR_ vs VMI_190keV_	0.997	1.00	0.984	1.00	1.00	1.00
Mixed_iMAR_ vs VMI_190keV-iMAR_	1.00	1.00	1.00	1.00	1.00	1.00
VMI_50keV-iMAR_ vs Mixed	< 0.001	0.021	0.004	1.00	0.083	1.00
VMI_50keV-iMAR_ vs VMI_190keV_	1.00	1.00	0.569	0.114	0.788	1.00
VMI_50keV-iMAR_ vs VMI_190keV-iMAR_	1.00	1.00	1.00	0.074	0.94	1.00

Corrected quantitative artifact as mean ± standard deviation in HU in patients with spinal implants, for image reconstructions which were also rated for subjective image quality. A complete table with VMI reconstructions for all keV steps can be found the supplementary material (supplementary Table 2b). Comparisons for displayed p-values were made with one-way ANOVA with Tukey honestly significant difference post-hoc test

*IVC* Inferior vena cava

Mixed_iMAR_ images showed significantly better artifact reduction compared to Mixed images without iMAR in hypodense artifacts in the abdominal aorta (*p* < 0.001), as well as in the hypodense artifact in subcutaneous tissue (*p* = 0.04). Between Mixed_iMAR_ and VMI_190keV-iMAR_ no significant difference in artifact reduction was observed in any ROI.

When comparing VMI_50keV-iMAR_ images to Mixed images without iMAR, VMI_50keV-iMAR_ showed significantly lower artifact in all hypodense artifacts. When comparing VMI_50keV-iMAR_ to Mixed_iMAR_ or VMI_190keV-iMAR_, no statistically significant difference in artifact severity was observed (all *p* > 0.05).

**Fig. 4:**
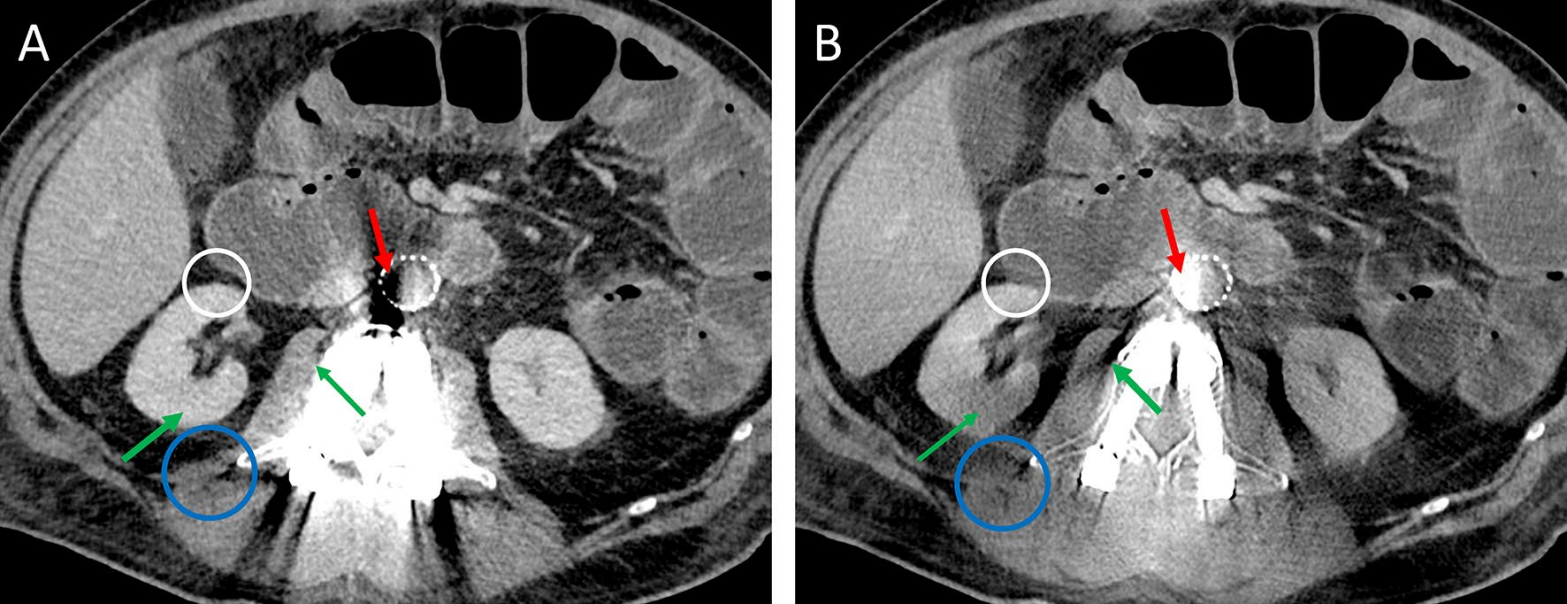
Axial images of a patient with spinal implants, window setting: window width 300 HU, window level 40 HU. **a** Mixed **b** Mixed_iMAR_. Overcorrection of an originally hypodense artifact into a hyperdense artifact in the aorta (red arrow). Additional new hypodense artifacts in the kidney and the psoas muscle (green arrows) and reduced organ margin sharpness between the duodenum and right kidney (white circle) or at the dorsal retroperitoneum (blue circle)

Overcorrection was seen in iMAR images in the hyperdense artifact in IVC and kidney as displayed in Fig. 4 and quantitatively shown in Fig. 3c and d.

### Image noise

For both hip and spinal implants in images without iMAR no significant difference in corrected image noise was observed between Mixed and VMI_190keV_ in all ROIs (all *p* = 1.00) (Supplementary Table 3a, b). Mixed_iMAR_ showed significantly lower corrected image noise compared to Mixed images in most tissues, for hip implants but not for spinal implants. VMI_190keV-iMAR_ did not significantly reduce corrected image noise compared to the Mixed_iMAR_ images (all *p* = 1.00) for both implant types.

### Subjective image quality

Overall interrater-agreement was good: ICC = 0.77 (95% Confidence-Interval: 0.71–0.82).

#### Hip implants

iMAR reconstructions were rated better than corresponding images without iMAR for overall image quality (Supplementary Table 4a), as well as all other diagnostic criteria.

**Fig. 5:**
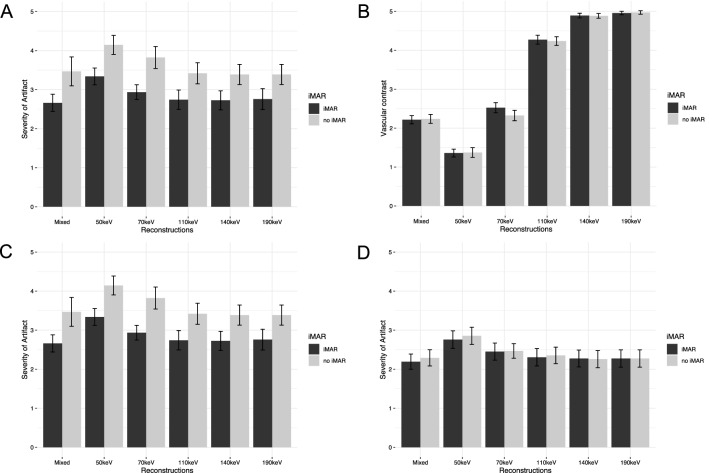
Subjective overall image quality for hip (**a**) and spinal (**c**) implants. Lower values describe lower artifact, see also supplementary Table 1. Subjective vascular contrast for hip implants (**b**). Subjective organ margin sharpness for spinal implants (**d**). Lower values describe better image quality/vascular contrast (see supplementary Table 4). Central bar shows the median, lower and upper hinges of the box correspond to the first and third quartiles, whiskers extend from the hinge to the largest value no further than 1.5 times the interquartile range

For overall diagnostic image quality Mixed_iMAR_ was rated best (Fig. 5a), and was significantly better than all other images with and without iMAR (for all *p* < 0.002). There was no significant difference between VMI_70keV-iMAR_ to VMI_190keV-iMAR_ (for all *p* > 0.07), suggesting that overall diagnostic image quality wasn’t further improved using higher keVs.

The evaluation of vascular contrast showed no significant difference between VMI images of the same keV level with and without iMAR (for all *p* > 0.52), except for VMI_70keV_ (*p* = 0.03) (Fig. 5b).

**Fig. 6:**
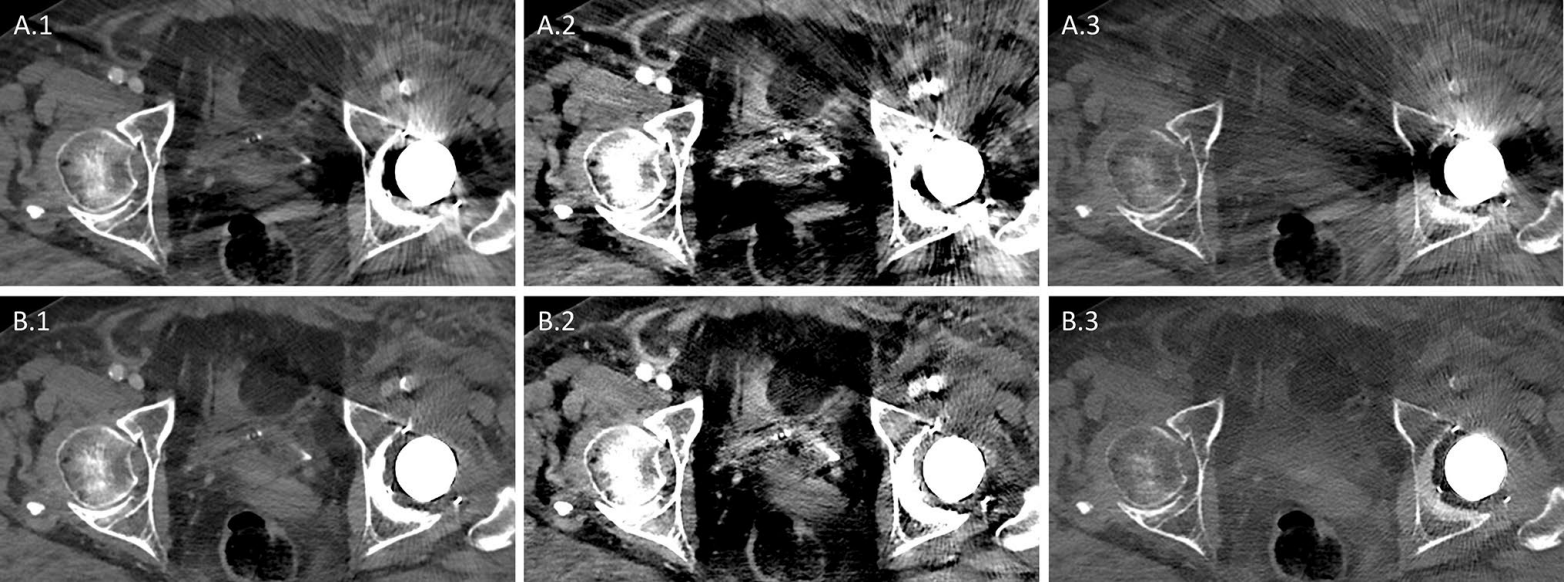
Axial images of a patient with unilateral hip implant. Images all shown with the same windowing: window width 600 HU, window level 150 HU. Images A without iMAR, images B with iMAR: (1) Mixed (2) VMI_50keV_ (3) VMI_190keV_. Note the improved vascular contrast on VMI_50keV-iMAR_ (A.2 and B.2) and the reduced tissue contrast on VMI_190keV-iMAR_ (A.3 and B.3)

While for overall image quality VMI_50keV-iMAR_ images were rated worse than Mixed_iMAR_ images and VMI_iMAR_ of higher keV levels, VMI_50keV-iMAR_ images were rated significantly better than images without iMAR, both Mixed and VMI of any keV level (for all *p* < 0.001). Additionally, VMI_50keV-iMAR_ were rated best in terms of visualization of vascular contrast (Fig. 6). They were rated significantly better than Mixed or VMI of all other keV levels with iMAR (for all *p* < 0.001).

#### Spinal implants

Mixed_iMAR_ and VMI_iMAR_ images were rated better in terms of overall diagnostic image quality (Fig. 5c), as well as muscle, osseous and prevertebral structure evaluation, compared to the corresponding images without iMAR (Supplementary Table 4b).

VMI_50keV-iMAR_ was not rated inferior than Mixed, VMI_110keV_, VMI_140keV_ and VMI_190keV_ images without iMAR (all *p* > 0.51) regarding overall diagnostic image quality.

While in the category organ margin sharpness, iMAR images were not rated better than corresponding images without iMAR (all *p* > 0.77) (Fig. 5d), for prevertebral structures iMAR images were significantly better than images without iMAR (all *p* < 0.005).

For the assessment of vascular contrast VMI_50keV-iMAR_ and VMI_50keV_ were rated better than all other images (all *p* < 0.001).

#### Patients with artifact at the site of clinical question

Thirty-two studies with hip implants had artifact in the area of interest. Subjective overall image quality and diagnostic quality improved from the Mixed images: 3.78 ± 0.98 to 1.67 ± 0.67 for the Mixed_iMAR_ images, *p* < 0.001.

For the six patients with spinal implants image quality improved from Mixed to Mixed_iMAR_ from 3.43 ± 1.5 to 2.29 ± 0.73, *p* = 0.006.

## Discussion

In our study we assessed the image quality and value of a dedicated iMAR together with VMI of sfDECT in abdominal CT of patients with hip or spinal implants. We found that both for hip and spinal implants Mixed_iMAR_ images are preferred. They showed quantitative artifact reduction in both hypo- and hyperdense artifact, as well as improved subjective image quality over Mixed images without iMAR in all diagnostic criteria. Additional high keV VMI_iMAR_ did not further improve image quality over Mixed_iMAR_. However, due to lower artifact on iMAR images, it is possible to use low keV images (VMI_50keV_) to improve vascular and soft tissue contrast.

Many previous works have evaluated iterative metal artifact reduction algorithms and high keV VMI for reducing artifacts in hip and spinal implants both in phantom and patient studies. However, studies that combined both iMAR algorithms and VMI had different outcomes depending on the algorithm and DECT platform used. In a phantom with hip implants, using both dual-source and rapid-kVp-switching DECT, Andersson et al. showed that iMAR images are preferred in the visual analysis over VMI [43], while quantitative artifact was lower when both approaches were combined [44]. In a patient study with hip implants Youe et al. found the combination of iMAR and VMI to be providing lowest artifact on rapid-kVp-switching DECT scanners [45]. Same was found for dual-layer spectral-detector DECT [24]. Bongers et al. could show similar results for dual-source DECT, but noted only an incremental value of adding high keV VMI to iMAR [46].

In contrast to these findings in hip implants, for spinal implants Wang et al. reported a preference for VMI over iMAR images when using rapid-kVp-switching DECT, due to massive overcorrection of artifacts on iMAR images [27]. Yet, for dual-source and dual-layer spectral-detector DECT the combination of iMAR and VMI was rated best [8, 25]. The only study on metal artifact reduction in sfDECT with iMAR investigated dental hardware in head and neck CT [47]. They found a greater impact of iMAR than VMI and only a slight benefit of combining both techniques.

While the majority of studies with hip implants preferred a combination of VMI and iMAR, our study indicates that for sfDECT Mixed_iMAR_ images are preferred. Despite not improving image quality, VMI_190keV-iMAR_ still showed lowest quantitative artifact. This is in line with the results of Bongers et al.[46], where only an incremental value of VMI to iMAR was seen. The inferior spectral separation of sfDECT compared to dual-source DECT may explain why in our study this small increase in artifact reduction with VMI did not provide enough additional benefit to be perceived helpful by the readers. For spinal implants readers also preferred Mixed_iMAR_ images in our study. This seems to be different from dual-source and dual-layer spectral-detector CT. Similarly to the findings in rapid-kVp-switching DECT and other studies [47, 48], overcorrection of artifact was also observed in spinal implants in our study when using iMAR, however to a much lesser degree. This may be because the iMAR algorithm, used in this study, combines beam hardening correction, normalized sinogram inpainting and frequency split, to address both, the avoidance of new artifacts and the conservation of the original image impression [5, 49]. Our study found low keV images with iMAR (VMI_50keV-iMAR_) increased vascular contrast, while maintaining sufficient image quality, which is of special interest in abdominal imaging. This differs from the findings of the only previous study that assessed iMAR and low keV VMI [47]. This may be due to the fact, that we investigated abdominal CT examinations instead of head and neck CT, with a different radiation dose and field of view.

A subgroup analysis of our patient cohort revealed that over a third of patients (37%) had implant-based metal artifact directly at the site of clinical question. Subjective image quality improved significantly from markedly reduced diagnostic interpretability to only slight artifacts without impaired diagnostic interpretability. This underlines the clinical importance of this technique to reduce image artifacts.

There are several limitations to this study. First this was a retrospective design. Second, readers for subjective image quality were not blinded toward the image reconstruction. iMAR reconstructions show a typical and recognizable appearance and have lower artifact, making a blinding of the readers not feasible. To assess objective artifact reduction the corrected HU were measured. While this allowed a fair comparison between mixed images and VMI, it is not used clinically. We did not distinguish between unilateral and bilateral hip prosthesis. Previous studies showed that MAR-algorithms especially yielded benefits over VMI alone in severe artifacts such as in bilateral hip prosthesis [50]. The results of our study are specific for split-filter dual-energy CT with the use of iMAR for hip and spinal implants, they do not transfer to other dual-energy CT scanner types or iterative metal artifact algorithms.

## Conclusion

For abdominal split-filter DECT of patients with spinal or hip implants iMAR should be used to minimize metal artifacts. Mixed_iMAR_ images provide best image quality. While high keV VMI can reduce quantitative artifact, they do not improve overall image quality. Low keV images (VMI_50keV_) may be used together with iMAR to improve vascular contrast, while providing less metal artifact compared to non iMAR images.

## Supplementary Information

Below is the link to the electronic supplementary material.

Supplementary file1 (DOCX 51 KB)

## References

[1] Boas FE, Fleischmann D (2012). CT artifacts: Causes and reduction techniques. Imaging Med.

[2] Andersson KM, Dahlgren CV, Reizenstein J (2018). Evaluation of two commercial CT metal artifact reduction algorithms for use in proton radiotherapy treatment planning in the head and neck area. Med Phys.

[3] Bolstad K, Flatabø S, Aadnevik D (2018). Metal artifact reduction in CT, a phantom study: subjective and objective evaluation of four commercial metal artifact reduction algorithms when used on three different orthopedic metal implants. Acta Radiol.

[4] Kidoh M, Nakaura T, Nakamura S (2014). Reduction of dental metallic artefacts in CT: Value of a newly developed algorithm for metal artefact reduction (O-MAR). Clin Radiol.

[5] Maerz M, Mittermair P, Krauss A (2016). Iterative metal artifact reduction improves dose calculation accuracy. Strahlentherapie und Onkol.

[6] Kim YJ, Cha JG, Kim H (2019). Dual-energy and iterative metal artifact reduction for reducing artifacts due to metallic hardware: A loosening hip phantom study. Am J Roentgenol.

[7] Toso S, Laurent M, Lozeron ED (2018). Iterative algorithms for metal artifact reduction in children with orthopedic prostheses: preliminary results. Pediatr Radiol.

[8] Long Z, Bruesewitz MR, DeLone DR (2018). Evaluation of projection- and dual-energy-based methods for metal artifact reduction in CT using a phantom study. J Appl Clin Med Phys.

[9] Katsura M, Sato J, Akahane M (2018). Current and novel techniques for metal artifact reduction at CT: Practical guide for radiologists. RadioGraphics.

[10] Siegel MJ, Kaza RK, Bolus DN (2017). White paper of the society of computed body tomography and magnetic resonance on dual-energy CT, part 3: Vascular, cardiac, pulmonary, and musculoskeletal applications. J Comput Assist Tomogr.

[11] Bamberg F, Dierks A, Nikolaou K (2011). Metal artifact reduction by dual energy computed tomography using monoenergetic extrapolation. Eur Radiol.

[12] Lewis M, Reid K, Toms AP (2013). Reducing the effects of metal artefact using high keV monoenergetic reconstruction of dual energy CT (DECT) in hip replacements. Skeletal Radiol.

[13] Siegel MJ, Kaza RK, Bolus DN (2016). White paper of the society of computed body tomography and magnetic resonance on dual-energy CT, part 1: Technology and terminology. J Comput Assist Tomogr.

[14] Zhou C, Zhao YE, Luo S (2011). Monoenergetic imaging of dual-energy CT reduces artifacts from implanted metal orthopedic devices in patients with factures. Acad Radiol.

[15] Meinel FG, Bischoff B, Zhang Q (2012). Metal artifact reduction by dual-energy computed tomography using energetic extrapolation: A systematically optimized protocol. Invest Radiol.

[16] Guggenberger R, Winklhofer S, Osterhoff G (2012). Metallic artefact reduction with monoenergetic dual-energy CT: Systematic ex vivo evaluation of posterior spinal fusion implants from various vendors and different spine levels. Eur Radiol.

[17] Filograna L, Magarelli N, Leone A (2015). Value of monoenergetic dual-energy CT (DECT) for artefact reduction from metallic orthopedic implants in post-mortem studies. Skeletal Radiol.

[18] Higashigaito K, Angst F, Runge VM (2015). Metal artifact reduction in pelvic computed tomography with hip prostheses. Invest Radiol.

[19] Komlosi P, Grady D, Smith JS (2015). Evaluation of monoenergetic imaging to reduce metallic instrumentation artifacts in computed tomography of the cervical spine. J Neurosurg Spine.

[20] Dong Y, Shi AJ, Wu JL (2016). Metal artifact reduction using virtual monochromatic images for patients with pedicle screws implants on CT. Eur Spine J.

[21] Wellenberg RHH, Hakvoort ET, Slump CH (2018). Metal artifact reduction techniques in musculoskeletal CT-imaging. Eur J Radiol.

[22] Neuhaus V, Große Hokamp N, Abdullayev N (2017). Metal artifact reduction by dual-layer computed tomography using virtual monoenergetic images. Eur J Radiol.

[23] Laukamp KR, Zopfs D, Lennartz S (2019). Metal artifacts in patients with large dental implants and bridges: Combination of metal artifact reduction algorithms and virtual monoenergetic images provides an approach to handle even strongest artifacts. Eur Radiol.

[24] Neuhaus V, Grosse Hokamp N, Zopfs D (2019). Reducing artifacts from total hip replacements in dual layer detector CT: Combination of virtual monoenergetic images and orthopedic metal artifact reduction. Eur J Radiol.

[25] Park J, Kim SH, Han JK (2019). Combined application of virtual monoenergetic high keV images and the orthopedic metal artifact reduction algorithm (O-MAR): effect on image quality. Abdom Radiol.

[26] Lee YH, Park KK, Song H-T (2012). Metal artefact reduction in gemstone spectral imaging dual-energy CT with and without metal artefact reduction software. Eur Radiol.

[27] Wang Y, Qian B, Li B (2013). Metal artifacts reduction using monochromatic images from spectral CT: Evaluation of pedicle screws in patients with scoliosis. Eur J Radiol.

[28] De Crop A, Casselman J, Van Hoof T (2015). Analysis of metal artifact reduction tools for dental hardware in CT scans of the oral cavity: kVp, iterative reconstruction, dual-energy CT, metal artifact reduction software: does it make a difference?. Neuroradiology.

[29] Han SC, Chung YE, Lee YH (2014). Metal artifact reduction software used with abdominopelvic dual-energy CT of patients with metal hip prostheses: Assessment of image quality and clinical feasibility. Am J Roentgenol.

[30] Euler A, Parakh A, Falkowski AL (2016). Initial results of a single-source dual-energy computed tomography technique using a split-filter: Assessment of image quality, radiation dose, and accuracy of dual-energy applications in an in vitro and in vivo study. Invest Radiol.

[31] Euler A, Obmann MM, Szucs-farkas Z (2018). Comparison of image quality and radiation dose between split-filter dual-energy images and single-energy images in single-source abdominal CT. Eur Radiol.

[32] Almeida IP, Schyns LEJR, Öllers MC (2017). Dual-energy CT quantitative imaging: A comparison study between twin-beam and dual-source CT scanners. Med Phys.

[33] Lennartz S, Laukamp KR, Tandon Y (2021). Abdominal vessel depiction on virtual triphasic spectral detector CT: Initial clinical experience. Abdom Radiol.

[34] Yoo J, Lee JM, Yoon JH (2021). Comparison of low kVp CT and dual-energy CT for the evaluation of hypervascular hepatocellular carcinoma. Abdom Radiol.

[35] Lourenco PDM, Rawski R, Mohammed MF (2018). Dual-energy CT iodine mapping and 40-keV monoenergetic applications in the diagnosis of acute bowel ischemia. Am J Roentgenol.

[36] Obmann MM, Punjabi G, Obmann VC (2021). Dual-energy CT of acute bowel ischemia. Abdom Radiol.

[37] Obmann MM, An C, Schaefer A (2020). Improved sensitivity and reader confidence in CT colonography using dual-layer spectral CT: A phantom study. Radiology.

[38] Anastasopoulos C, Reisert M, Kellner E (2017). “Nora Imaging”: A web-based platform for medical imaging. Neuropediatrics.

[39] Große Hokamp N, Neuhaus V, Abdullayev N (2017). Reduction of artifacts caused by orthopedic hardware in the spine in spectral detector CT examinations using virtual monoenergetic image reconstructions and metal-artifact-reduction algorithms. Skeletal Radiol.

[40] Fleiss JL, Cohen J (1973). The equivalence of weighted kappa and the intraclass correlation coefficient as measures of reliability. Educ Psychol Meas.

[41] Koo TK, Li MY (2016). A guideline of selecting and reporting intraclass correlation coefficients for reliability research. J Chiropr Med.

[42] R Core Team (2018) R: A language and environment for statistical computing. R Foundation for statistical Computing, 2018, Vienna, Austria. https://www.r-project.org/. Accessed 22 Sep 2022

[43] Andersson KM, Norrman E, Geijer H (2016). Visual grading evaluation of commercially available metal artefact reduction techniques in hip prosthesis computed tomography. Br J Radiol.

[44] Andersson KM, Nowik P, Persliden J (2015). Metal artefact reduction in CT imaging of hip prostheses—an evaluation of commercial techniques provided by four vendors. Br J Radiol.

[45] Yue D, Fan Rong C, Ning C (2018). Reduction of metal artifacts from unilateral hip arthroplasty on dual-energy CT with metal artifact reduction software. Acta Radiol.

[46] Bongers MN, Schabel C, Thomas C (2015). Comparison and combination of dual-energy- and iterative-based metal artefact reduction on hip prosthesis and dental implants. PLoS One.

[47] Schmidt AMA, Grunz J-P, Petritsch B (2021). Combination of iterative metal artifact reduction and virtual monoenergetic reconstruction using split-filter dual-energy CT in patients with dental artifact on head and neck CT. Am J Roentgenol.

[48] Pettersson E, Bäck A, Thilander-Klang A (2021). Comparison of metal artefacts for different dual energy CT techniques. Radiat Prot Dosimetry.

[49] Kachelrieß M, Krauss A (2015). Iterative metal artifact reduction (iMAR): Technical principles and clinical results in radiation therapy.

[50] Laukamp KR, Lennartz S, Neuhaus VF (2018). CT metal artifacts in patients with total hip replacements: For artifact reduction monoenergetic reconstructions and post-processing algorithms are both efficient but not similar. Eur Radiol.

