# Supplementary material for: Metal implants on abdominal CT: does split-filter dual-energy CT provide additional value over iterative metal artifact reduction?
Source: Abdom Radiol (NY). 2022 Sep 30;48(1):424–35. doi: 10.1007/s00261-022-03682-3 (PMC9849167; doi:10.1007/s00261-022-03682-3)
Supplement: Supplementary file 1 — Supplementary file1 (DOCX 51 KB) [file 261_2022_3682_MOESM1_ESM.docx]

Supplementary Material

Supplementary Table 1

| **Score** | **Overall diagnostic image quality** | **Bone** | **Adjacent muscle, soft tissue** | **Bladder wall &  rectal wall** | **Vascular contrast** | **Organ margin sharpness and prevertebral structures (spine only)** |
| --- | --- | --- | --- | --- | --- | --- |
| **1** | Subtle artifacts,  full diagnostic quality | Subtle artifacts, but bone is fully visible, inhomogeneities in the trabecular bone may be present | Subtle artifacts | Subtle artifacts, bladder and rectal wall are clearly definable and can be distinguished from adjacent structures | Excellent contrast | Subtle artifacts/ Excellent image quality |
| **2** | Mild artifacts, but  diagnostic interpretability is  not impaired | Mild artifacts, inhomogeneities in trabecular bone stronger than in 1, but no impairment by streaks | Mild artifacts | Mild artifacts, <25% of one side of the wall is impaired by artifacts | Very good contrast | Mild artifacts/ Very good image quality |
| **3** | Moderate artifacts, slightly impaired diagnostic interpretability | Moderate artifacts, trabecular bone is impaired by streaks | Moderate artifacts | Moderate artifacts, 25-50% of one side of the wall is impaired by artifacts (or in total if artifacts impair more than one side of the wall) | Good contrast | Moderate artifacts/ Good or acceptable image quality |
| **4** | Severe artifacts, markedly reduced diagnostic interpretability | Severe artifacts, streaks stronger than in 3, cortical bone is slightly impaired by streaks | Severe artifacts | Severe artifacts, 50-75% of one side of the wall is impaired by artifacts (or in total if artifacts impair more than one side of the wall) | Poor contrast | Severe artifacts/ Poor image quality |
| **5** | Massive artifacts, insufficient diagnostic quality | Massive artifacts, cortical bone is partially indiscriminable | Massive artifacts | Massive artifacts, >75% of one side of the wall are not visible due to the artifact (or in total if artifacts impair more than one side of the wall) | Images similar to the use of no contrast | Massive artifacts/ Insufficient image quality |

Suppl. Table 1: Further specification of evaluation criteria for the readers

Supplementary Table 2

a) Quantitative artifact in hip implants

|  | Muscle  hyperdense artifact | Muscle hypodense artifact | Subcutan  hyperdense artifact | Subcutan  hypodense artifact | Bladder  hyperdense artifact | Bladder  hypodense artifact |
| --- | --- | --- | --- | --- | --- | --- |
| Mixed | 206.51 ± 105.16 | -260.05 ± 141.4 | 52.74 ± 29.37 | -133.54 ± 77.54 | 85.16 ± 95.85 | -180.31 ± 139.37 |
| VMI_40keV_ | 641.42 ± 599.94 | -660.46 ± 349.86 | 161.64 ± 183.46 | -439.52 ± 230.87 | 244.16 ± 380.41 | -516.87 ± 369.97 |
| VMI_50keV_ | 453.92 ± 366.29 | -526.81 ± 293.63 | 113.82 ± 112.48 | -321.6 ± 180.64 | 174.62 ± 248.51 | -412.61 ± 320.33 |
| VMI_60keV_ | 340.75 ± 230.24 | -411.14 ± 224.71 | 84.88 ± 70.65 | -237.34 ± 130.52 | 133.64 ± 171.68 | -316.15 ± 252.03 |
| VMI_70keV_ | 271.61 ± 155.68 | -333.55 ± 176.31 | 67.05 ± 46.65 | -184.79 ± 100.81 | 108.88 ± 129.13 | -247.03 ± 194.23 |
| VMI_80keV_ | 226.84 ± 118.51 | -283.48 ± 152.1 | 55.69 ± 33.64 | -151.08 ± 84.11 | 93.32 ± 105.95 | -201.68 ± 157.89 |
| VMI_90keV_ | 196.91 ± 104.59 | -249.68 ± 141.1 | 48.13 ± 28.07 | -128.48 ± 74.84 | 82.55 ± 93.13 | -170.92 ± 134.7 |
| VMI_100keV_ | 175.58 ± 102.34 | -226.24 ± 135.92 | 42.66 ± 26.5 | -112.87 ± 69.76 | 75.25 ± 86.5 | -149.21 ± 120.28 |
| VMI_110keV_ | 160.6 ± 105.13 | -209.74 ± 134.02 | 38.84 ± 26.97 | -101.81 ± 66.99 | 70.05 ± 82.79 | -133.82 ± 111.07 |
| VMI_120keV_ | 149.73 ± 109.19 | -197.69 ± 133.6 | 36.06 ± 28.07 | -93.75 ± 65.4 | 66.24 ± 80.78 | -122.58 ± 105.05 |
| VMI_130keV_ | 141.68 ± 113.13 | -188.73 ± 133.79 | 33.97 ± 29.26 | -87.74 ± 64.47 | 63.38 ± 79.69 | -114.21 ± 101.09 |
| VMI_140keV_ | 135.67 ± 116.35 | -181.9 ± 134.28 | 32.39 ± 30.36 | -83.15 ± 63.94 | 61.2 ± 79.11 | -107.85 ± 98.4 |
| VMI_150keV_ | 131.06 ± 119.02 | -176.62 ± 134.81 | 31.16 ± 31.3 | -79.6 ± 63.62 | 59.49 ± 78.79 | -102.93 ± 96.55 |
| VMI_160keV_ | 127.41 ± 121.32 | -172.45 ± 135.31 | 30.2 ± 32.09 | -76.81 ± 63.44 | 58.15 ± 78.66 | -99.05 ± 95.19 |
| VMI_170keV_ | 124.4 ± 123.23 | -169.1 ± 135.82 | 29.41 ± 32.77 | -74.56 ± 63.35 | 57.08 ± 78.6 | -95.97 ± 94.2 |
| VMI_180keV_ | 122.12 ± 124.93 | -166.5 ± 136.29 | 28.82 ± 33.32 | -72.8 ± 63.27 | 56.23 ± 78.62 | -93.47 ± 93.45 |
| VMI_190keV_ | 120.17 ± 126.32 | -164.33 ± 136.68 | 28.3 ± 33.79 | -71.33 ± 63.23 | 55.53 ± 78.65 | -91.44 ± 92.89 |
| Mixed_iMAR_ | 2.59 ± 29.14 | -18.37 ± 69.7 | 16.45 ± 18.9 | 3.71 ± 18.68 | 4.45 ± 20.13 | -12.09 ± 16.89 |
| VMI_40keV-iMAR_ | -15.26 ± 208.05 | -39.41 ± 321.04 | 45.71 ± 132.45 | -8.28 ± 99.18 | 24.14 ± 87.45 | -19.99 ± 79.25 |
| VMI_50keV-iMAR_ | -7.22 ± 126.6 | -30.55 ± 212.37 | 31.99 ± 79.37 | -4.52 ± 60.64 | 15.73 ± 55.84 | -16.8 ± 50.31 |
| VMI_60keV-iMAR_ | -2.35 ± 78.2 | -24.7 ± 146.74 | 23.74 ± 48.03 | -2.15 ± 38.34 | 10.6 ± 37.47 | -14.72 ± 33.51 |
| VMI_70keV-iMAR_ | 0.69 ± 50.18 | -21.24 ± 106.46 | 18.71 ± 30.25 | -0.73 ± 26.33 | 7.33 ± 27.31 | -13.43 ± 24.01 |
| VMI_80keV-iMAR_ | 2.19 ± 34.67 | -18.77 ± 82.18 | 15.5 ± 21.1 | 0.31 ± 20.67 | 5.33 ± 21.92 | -12.5 ± 18.75 |
| VMI_90keV-iMAR_ | 3.23 ± 27.73 | -17.09 ± 66.86 | 13.44 ± 17.94 | 1 ± 18.8 | 3.96 ± 19.2 | -11.85 ± 16.2 |
| VMI_100keViMAR_ | 3.88 ± 25.99 | -15.71 ± 57.01 | 11.81 ± 17.9 | 1.41 ± 18.83 | 3.01 ± 17.93 | -11.52 ± 15.09 |
| VMI_110keV-iMAR_ | 4.38 ± 26.78 | -14.83 ± 50.72 | 10.69 ± 19.12 | 1.72 ± 19.51 | 2.3 ± 17.47 | -11.24 ± 14.75 |
| VMI_120keV-iMAR_ | 4.76 ± 28.35 | -14.19 ± 46.6 | 9.87 ± 20.57 | 1.94 ± 20.31 | 1.81 ± 17.39 | -11.04 ± 14.76 |
| VMI_130keV-iMAR_ | 5.06 ± 29.94 | -13.74 ± 43.85 | 9.26 ± 21.88 | 2.13 ± 21.02 | 1.44 ± 17.45 | -10.89 ± 14.88 |
| VMI_140keV-iMAR_ | 5.28 ± 31.39 | -13.39 ± 41.97 | 8.79 ± 22.98 | 2.29 ± 21.63 | 1.16 ± 17.57 | -10.78 ± 15.04 |
| VMI_150keV-iMAR_ | 5.44 ± 32.6 | -13.13 ± 40.67 | 8.44 ± 23.9 | 2.4 ± 22.16 | 0.94 ± 17.71 | -10.7 ± 15.21 |
| VMI_160keV-iMAR_ | 5.57 ± 33.61 | -12.92 ± 39.74 | 8.17 ± 24.64 | 2.48 ± 22.59 | 0.78 ± 17.84 | -10.63 ± 15.36 |
| VMI_170keV-iMAR_ | 5.68 ± 34.46 | -12.76 ± 39.06 | 7.96 ± 25.25 | 2.56 ± 22.94 | 0.64 ± 17.97 | -10.58 ± 15.51 |
| VMI_180keV-iMAR_ | 5.77 ± 35.16 | -12.63 ± 38.58 | 7.78 ± 25.75 | 2.61 ± 23.24 | 0.53 ± 18.08 | -10.53 ± 15.63 |
| VMI_190keV-iMAR_ | 5.84 ± 35.74 | -12.52 ± 38.2 | 7.64 ± 26.18 | 2.66 ± 23.49 | 0.44 ± 18.17 | -10.5 ± 15.74 |

Suppl. Table 2a: Corrected quantitative artifact as mean± standard deviation in HU for all measured ROIs in patients with hip implants.

b) Quantitative artifact in spinal implants

|  | Abdominal aorta hypodense artifact | IVC hyperdense artifact | Subcutan hypodense artifact | Subcutan hyperdense artifact | Kidney hyperdense artifact | Psoas muscle hyperdense artifact |
| --- | --- | --- | --- | --- | --- | --- |
| Mixed | -304.11 ± 166.93 | 56.14 ± 45.35 | -85.46 ± 66.91 | 77.64 ± 53.02 | 17.55 ± 31.95 | 77.5 ± 88.49 |
| VMI_40keV_ | -1023.33 ± 427.06 | 270.11 ± 248.49 | -226.95 ± 201.18 | 311.78 ± 238.3 | 91.81 ± 176.16 | 398.18 ± 353.42 |
| VMI_50keV_ | -727.41 ± 323.81 | 176.25 ± 157.45 | -164.14 ± 136.42 | 209.4 ± 152.81 | 60.15 ± 112.43 | 258.19 ± 236.38 |
| VMI_60keV_ | -524.78 ± 245.21 | 119.66 ± 102.57 | -127.29 ± 99.53 | 147.25 ± 102.42 | 41.12 ± 74.06 | 172.92 ± 166.4 |
| VMI_70keV_ | -403.86 ± 201.29 | 85.3 ± 70.35 | -104.71 ± 79.02 | 108.76 ± 73.29 | 29.52 ± 51.24 | 120.74 ± 124.89 |
| VMI_80keV_ | -322.53 ± 174.68 | 62.98 ± 50.88 | -89.73 ± 68.31 | 83.48 ± 56.54 | 21.46 ± 37 | 86.85 ± 98.45 |
| VMI_90keV_ | -268.24 ± 158.91 | 47.02 ± 40.25 | -79.43 ± 62.34 | 65.82 ± 46.8 | 15.9 ± 27.85 | 63.49 ± 81.24 |
| VMI_100keV_ | -230.81 ± 151.05 | 35.67 ± 34.91 | -72.36 ± 59.44 | 53.74 ± 42.1 | 11.95 ± 22.19 | 46.92 ± 69.83 |
| VMI_110keV_ | -204.14 ± 145.95 | 27.75 ± 32.99 | -67.31 ± 57.59 | 45.31 ± 40.08 | 9.24 ± 19.01 | 35.31 ± 62.36 |
| VMI_120keV_ | -184.66 ± 142.67 | 22.01 ± 32.73 | -63.65 ± 56.45 | 39.16 ± 39.41 | 7.31 ± 17.38 | 26.88 ± 57.28 |
| VMI_130keV_ | -170.15 ± 140.53 | 17.72 ± 33.17 | -60.91 ± 55.75 | 34.58 ± 39.41 | 5.86 ± 16.64 | 20.61 ± 53.75 |
| VMI_140keV_ | -159.09 ± 139.1 | 14.48 ± 33.88 | -58.81 ± 55.3 | 31.12 ± 39.66 | 4.76 ± 16.41 | 15.85 ± 51.24 |
| VMI_150keV_ | -150.54 ± 138.13 | 11.96 ± 34.63 | -57.21 ± 54.99 | 28.4 ± 40.01 | 3.91 ± 16.42 | 12.15 ± 49.42 |
| VMI_160keV_ | -143.83 ± 137.4 | 10 ± 35.34 | -55.95 ± 54.81 | 26.28 ± 40.37 | 3.25 ± 16.55 | 9.26 ± 48.08 |
| VMI_170keV_ | -138.45 ± 136.88 | 8.43 ± 35.96 | -54.94 ± 54.69 | 24.6 ± 40.67 | 2.72 ± 16.74 | 6.96 ± 47.07 |
| VMI_180keV_ | -134.14 ± 136.52 | 7.17 ± 36.53 | -54.14 ± 54.58 | 23.2 ± 40.95 | 2.3 ± 16.92 | 5.1 ± 46.29 |
| VMI_190keV_ | -130.62 ± 136.22 | 6.13 ± 37 | -53.47 ± 54.5 | 22.1 ± 41.25 | 1.94 ± 17.1 | 3.57 ± 45.68 |
| Mixed_iMAR_ | -54.55 ± 115.44 | -10.44 ± 32.39 | -8.63 ± 40.39 | 42.01 ± 38.06 | -13.53 ± 30.2 | 15.55 ± 51.14 |
| VMI_40keV-iMAR_ | -78.02 ± 314.56 | -31.32 ± 191.94 | 17.06 ± 181.27 | 132.54 ± 186.06 | -52.8 ± 113.75 | 64.63 ± 222.08 |
| VMI_50keV-iMAR_ | -70.28 ± 252.32 | -21.98 ± 119.93 | 2.69 ± 132.04 | 92.8 ± 115.88 | -34.47 ± 72.84 | 43.06 ± 145 |
| VMI_60keV-iMAR_ | -63.29 ± 198.64 | -16.08 ± 76.64 | -2.86 ± 87.35 | 68.93 ± 75.66 | -23.13 ± 49.73 | 29.83 ± 99.48 |
| VMI_70keV-iMAR_ | -57 ± 155.01 | -12.55 ± 50.95 | -6.34 ± 60.59 | 54.13 ± 53.07 | -16.43 ± 37.48 | 22.1 ± 72.92 |
| VMI_80keV-iMAR_ | -53.48 ± 123.77 | -10.09 ± 35.62 | -8.75 ± 44.3 | 44.17 ± 41.29 | -12.5 ± 32.01 | 17.25 ± 56.43 |
| VMI_90keV-iMAR_ | -50.64 ± 104.09 | -8.75 ± 27.19 | -10.15 ± 33.81 | 37.02 ± 35.61 | -9.6 ± 29.99 | 13.83 ± 46.86 |
| VMI_100keViMAR_ | -48.82 ± 91.24 | -7.98 ± 23.03 | -10.86 ± 27.61 | 32.05 ± 33.53 | -7.66 ± 29.67 | 11.29 ± 41.39 |
| VMI_110keV-iMAR_ | -47.36 ± 82.83 | -7.45 ± 21.77 | -11.42 ± 24.65 | 28.63 ± 33.27 | -6.26 ± 29.69 | 9.51 ± 38.32 |
| VMI_120keV-iMAR_ | -46.25 ± 77.17 | -7.02 ± 21.94 | -11.89 ± 23.52 | 26.17 ± 33.72 | -5.23 ± 29.9 | 8.27 ± 36.68 |
| VMI_130keV-iMAR_ | -45.49 ± 73.2 | -6.69 ± 22.6 | -12.22 ± 23.32 | 24.3 ± 34.4 | -4.49 ± 30.17 | 7.33 ± 35.8 |
| VMI_140keV-iMAR_ | -44.81 ± 70.37 | -6.43 ± 23.44 | -12.48 ± 23.49 | 22.9 ± 35.1 | -3.9 ± 30.43 | 6.62 ± 35.31 |
| VMI_150keV-iMAR_ | -44.27 ± 68.28 | -6.23 ± 24.22 | -12.7 ± 23.88 | 21.82 ± 35.74 | -3.47 ± 30.68 | 6.05 ± 35.06 |
| VMI_160keV-iMAR_ | -43.87 ± 66.7 | -6.09 ± 24.93 | -12.83 ± 24.26 | 20.98 ± 36.27 | -3.12 ± 30.9 | 5.62 ± 34.94 |
| VMI_170keV-iMAR_ | -43.56 ± 65.54 | -5.96 ± 25.57 | -12.97 ± 24.66 | 20.32 ± 36.76 | -2.84 ± 31.08 | 5.27 ± 34.91 |
| VMI_180keV-iMAR_ | -43.29 ± 64.62 | -5.84 ± 26.1 | -13.05 ± 25.01 | 19.74 ± 37.17 | -2.62 ± 31.24 | 4.99 ± 34.91 |
| VMI_190keV-iMAR_ | -43.1 ± 63.88 | -5.75 ± 26.55 | -13.14 ± 25.34 | 19.29 ± 37.5 | -2.43 ± 31.36 | 4.76 ± 34.93 |

Suppl. Table 2b: Corrected quantitative artifact as mean± standard deviation in HU for all measured ROIs in patients with spinal implants.

Supplementary Table 3

a) Standard deviation in hip implants

|  | Muscle  hyperdense artifact | Muscle hypodense artifact | Subcutan  hyperdense artifact | Subcutan  hypodense artifact | Bladder  hyperdense artifact | Bladder  hypodense artifact |
| --- | --- | --- | --- | --- | --- | --- |
| Mixed | 35.04 ± 21.32 | 26.54 + 23.83 | 7.42 ± 4.77 | 14.97 ± 17.24 | 10.62 ± 16.54 | 19.13 ± 29.69 |
| VMI_40keV_ | 151.56 ± 90.74 | 56.81 + 63.84 | 28.43 ± 20.81 | 56.26 ± 51.4 | 28.01 ± 54.95 | 7.92 ± 58.23 |
| VMI_50keV_ | 98.75 ± 58.18 | 55.37 + 48.87 | 19.55 ± 12.44 | 46.17 ± 38.74 | 20.44 ± 35.97 | 25.28 ± 42.41 |
| VMI_60keV_ | 68.19 ± 39.14 | 45.21 + 36.49 | 14.34 ± 7.98 | 32.36 ± 30.74 | 16.38 ± 26.17 | 28.35 ± 34.17 |
| VMI_70keV_ | 50.48 ± 28.59 | 35.92 + 29.94 | 11.13 ± 5.93 | 23.03 ± 23.31 | 13.74 ± 21.15 | 24.79 ± 33.61 |
| VMI_80keV_ | 39.95 ± 23.2 | 29.87 + 25.65 | 8.41 ± 4.82 | 17.42 ± 18.93 | 11.52 ± 17.76 | 21.28 ± 31.56 |
| VMI_90keV_ | 34.01 ± 21.07 | 25.66 + 22.97 | 7.65 ± 4.9 | 14.15 ± 15.77 | 10.49 ± 15.86 | 18.33 ± 28.2 |
| VMI_100keV_ | 31.93 ± 20.04 | 23.71 + 20.51 | 8.01 ± 5.12 | 12.33 ± 13.85 | 10.19 ± 14.52 | 16.06 ± 24.73 |
| VMI_110keV_ | 30.65 ± 19.87 | 22.44 + 19.3 | 8.38 ± 5.42 | 11.41 ± 12.62 | 10.06 ± 14.13 | 14.79 ± 23.12 |
| VMI_120keV_ | 29.81 ± 19.88 | 21.58 + 18.6 | 8.61 ± 5.65 | 10.87 ± 11.83 | 9.93 ± 13.91 | 13.94 ± 22.07 |
| VMI_130keV_ | 29.28 ± 19.98 | 21 + 18.15 | 8.78 ± 5.79 | 10.54 ± 11.29 | 9.84 ± 13.86 | 13.36 ± 21.46 |
| VMI_140keV_ | 28.89 ± 20.12 | 20.56 + 17.86 | 8.9 ± 5.89 | 10.33 ± 10.92 | 9.76 ± 13.79 | 12.95 ± 21.07 |
| VMI_150keV_ | 28.62 ± 20.25 | 20.24 + 17.62 | 9.02 ± 5.95 | 10.23 ± 10.64 | 9.73 ± 13.83 | 12.66 ± 20.81 |
| VMI_160keV_ | 28.44 ± 20.36 | 20 + 17.46 | 9.09 ± 6.01 | 10.15 ± 10.43 | 9.69 ± 13.85 | 12.41 ± 20.56 |
| VMI_170keV_ | 28.33 ± 20.45 | 19.83 + 17.33 | 9.17 ± 6.06 | 10.12 ± 10.27 | 9.65 ± 13.86 | 12.24 ± 20.38 |
| VMI_180keV_ | 28.26 ± 20.54 | 19.68 + 17.25 | 9.23 ± 6.1 | 10.09 ± 10.14 | 9.64 ± 13.87 | 12.1 ± 20.23 |
| VMI_190keV_ | 28.22 ± 20.6 | 19.57 + 17.17 | 9.28 ± 6.13 | 10.08 ± 10.04 | 9.62 ± 13.89 | 11.99 ± 20.13 |
| Mixed_iMAR_ | 8.94 ± 5.2 | 11.13 + 42.46 | 3.82 ± 4.96 | 3.08 ± 4.56 | 0.7 ± 4.14 | -0.48 ± 4.1 |
| VMI_40keV-iMAR_ | 45.53 ± 37.59 | 58.57 + 186.95 | 22.82 ± 19.84 | 18.05 ± 25.34 | 3.54 ± 15.19 | -4.4 ± 19.03 |
| VMI_50keV-iMAR_ | 29.94 ± 21.75 | 37.77 + 125.98 | 14.45 ± 11.22 | 10.97 ± 15.15 | 2.29 ± 9.9 | -1.48 ± 11.48 |
| VMI_60keV-iMAR_ | 20.64 ± 13.03 | 25.48 + 88.41 | 9.42 ± 6.91 | 6.91 ± 9.28 | 1.6 ± 7.27 | -0.13 ± 7.67 |
| VMI_70keV-iMAR_ | 14.93 ± 8.24 | 18.27 + 63.49 | 6.36 ± 5.07 | 4.66 ± 6.43 | 1.17 ± 5.7 | -0.03 ± 5.7 |
| VMI_80keV-iMAR_ | 10.64 ± 5.96 | 13.9 + 50.32 | 4.72 ± 4.9 | 3.74 ± 5.09 | 0.88 ± 4.41 | -0.39 ± 4.57 |
| VMI_90keV-iMAR_ | 9.51 ± 5.61 | 10.57 + 41.12 | 4 ± 4.92 | 3.11 ± 4.24 | 1 ± 4.07 | -0.37 ± 3.87 |
| VMI_100keViMAR_ | 9.48 ± 5.63 | 9.73 + 34.73 | 4.14 ± 5.01 | 2.76 ± 3.96 | 1.5 ± 4.18 | -0.64 ± 4.17 |
| VMI_110keV-iMAR_ | 9.47 ± 5.75 | 8.98 + 30.16 | 4.3 ± 5.11 | 2.85 ± 3.85 | 1.65 ± 4.42 | -0.69 ± 4.25 |
| VMI_120keV-iMAR_ | 9.45 ± 5.8 | 8.4 + 26.84 | 4.42 ± 5.18 | 2.95 ± 3.79 | 1.69 ± 4.55 | -0.75 ± 4.29 |
| VMI_130keV-iMAR_ | 9.44 ± 5.81 | 7.95 + 24.38 | 4.54 ± 5.24 | 3.1 ± 3.78 | 1.7 ± 4.63 | -0.79 ± 4.32 |
| VMI_140keV-iMAR_ | 9.43 ± 5.81 | 7.61 + 22.53 | 4.64 ± 5.29 | 3.21 ± 3.8 | 1.69 ± 4.71 | -0.82 ± 4.34 |
| VMI_150keV-iMAR_ | 9.44 ± 5.81 | 7.34 + 21.09 | 4.71 ± 5.34 | 3.29 ± 3.83 | 1.68 ± 4.75 | -0.84 ± 4.36 |
| VMI_160keV-iMAR_ | 9.45 ± 5.81 | 7.14 + 19.97 | 4.77 ± 5.38 | 3.35 ± 3.87 | 1.66 ± 4.79 | -0.87 ± 4.36 |
| VMI_170keV-iMAR_ | 9.45 ± 5.81 | 6.98 + 19.07 | 4.81 ± 5.4 | 3.41 ± 3.89 | 1.65 ± 4.82 | -0.88 ± 4.37 |
| VMI_180keV-iMAR_ | 9.47 ± 5.82 | 6.86 + 18.35 | 4.86 ± 5.42 | 3.46 ± 3.91 | 1.63 ± 4.85 | -0.91 ± 4.38 |
| VMI_190keV-iMAR_ | 9.48 ± 5.82 | 6.76 + 17.77 | 4.91 ± 5.44 | 3.49 ± 3.94 | 1.64 ± 4.87 | -0.92 ± 4.38 |

Suppl. Table 3a: Corrected image noise as mean± standard deviation for all measured ROIs in patients with hip implants.

b) Standard deviation in spinal implants

|  | Abdominal aorta hypodense artifact | IVC hyperdense artifact | Subcutan hypodense artifact | Subcutan hyperdense artifact | Kidney hyperdense artifact | Psoas muscle hyperdense artifact |
| --- | --- | --- | --- | --- | --- | --- |
| Mixed | 38,1 ± 27,99 | 10,96 ± 7,48 | 11,79 ± 10,03 | 8,42 ± 6,76 | 7,1 ± 6,71 | 19,81 ± 17,88 |
| VMI_40keV_ | 77,58 ± 63,09 | 61,09 ± 47,28 | 40,78 ± 37,56 | 37,15 ± 36,36 | 27,26 ± 31,46 | 104,75 ± 78,91 |
| VMI_50keV_ | 75,9 ± 54,29 | 39,48 ± 29,07 | 28,43 ± 24,08 | 24,88 ± 22,59 | 18,61 ± 20,04 | 66,9 ± 50,87 |
| VMI_60keV_ | 57,43 ± 36,86 | 25,48 ± 18,41 | 20,53 ± 16,58 | 17,47 ± 14,42 | 13,39 ± 13,46 | 44,09 ± 34,11 |
| VMI_70keV_ | 47,86 ± 31,51 | 17,33 ± 12,15 | 15,81 ± 12,93 | 12,96 ± 9,63 | 10,56 ± 9,59 | 30,79 ± 25,38 |
| VMI_80keV_ | 39,82 ± 27,76 | 12,75 ± 8,53 | 12,87 ± 10,09 | 9,42 ± 7,12 | 8,02 ± 7,43 | 22,53 ± 20,02 |
| VMI_90keV_ | 34,34 ± 27,21 | 10,32 ± 6,71 | 11 ± 8,57 | 7,96 ± 6,05 | 7,09 ± 6,74 | 17,97 ± 16,58 |
| VMI_100keV_ | 30,48 ± 27,03 | 10,09 ± 6,37 | 10,18 ± 7,81 | 7,72 ± 5,7 | 7,23 ± 7,21 | 16,28 ± 15,2 |
| VMI_110keV_ | 27,93 ± 27,75 | 9,74 ± 6,34 | 9,68 ± 7,47 | 7,51 ± 5,56 | 7,21 ± 7,33 | 15,17 ± 14,19 |
| VMI_120keV_ | 26,14 ± 28,41 | 9,45 ± 6,3 | 9,31 ± 7,33 | 7,3 ± 5,52 | 7,17 ± 7,36 | 14,48 ± 13,44 |
| VMI_130keV_ | 24,85 ± 29,01 | 9,26 ± 6,31 | 9,06 ± 7,29 | 7,15 ± 5,47 | 7,12 ± 7,33 | 14,03 ± 12,89 |
| VMI_140keV_ | 23,93 ± 29,51 | 9,11 ± 6,29 | 8,9 ± 7,32 | 7,03 ± 5,5 | 7,09 ± 7,3 | 13,74 ± 12,49 |
| VMI_150keV_ | 23,22 ± 29,92 | 9,01 ± 6,3 | 8,76 ± 7,34 | 6,93 ± 5,52 | 7,04 ± 7,27 | 13,51 ± 12,18 |
| VMI_160keV_ | 22,74 ± 30,26 | 8,93 ± 6,3 | 8,72 ± 7,38 | 6,89 ± 5,54 | 7,01 ± 7,24 | 13,36 ± 11,94 |
| VMI_170keV_ | 22,31 ± 30,54 | 8,87 ± 6,29 | 8,65 ± 7,38 | 6,86 ± 5,56 | 6,98 ± 7,21 | 13,24 ± 11,77 |
| VMI_180keV_ | 21,97 ± 30,78 | 8,83 ± 6,29 | 8,59 ± 7,4 | 6,8 ± 5,58 | 6,97 ± 7,19 | 13,15 ± 11,62 |
| VMI_190keV_ | 21,73 ± 30,97 | 8,8 ± 6,29 | 8,55 ± 7,43 | 6,76 ± 5,6 | 6,95 ± 7,16 | 13,09 ± 11,53 |
| Mixed_iMAR_ | 12,37 ± 24,49 | 7,61 ± 5,41 | 6,05 ± 7,02 | 7,59 ± 6,15 | 5,68 ± 4,36 | 12,87 ± 8,3 |
| VMI_40keV-iMAR_ | 13,13 ± 23,6 | 24,29 ± 29,11 | 11,56 ± 18,69 | 37,25 ± 25,84 | 20,73 ± 28,87 | 40,77 ± 34,06 |
| VMI_50keV-iMAR_ | 15,51 ± 32,18 | 18,16 ± 17,64 | 12,5 ± 20,63 | 23,84 ± 16,34 | 14,56 ± 17,6 | 28,78 ± 21,59 |
| VMI_60keV-iMAR_ | 16,06 ± 38,93 | 14,15 ± 11,58 | 9,9 ± 14,03 | 16,19 ± 11,27 | 10,89 ± 10,82 | 21,99 ± 14,88 |
| VMI_70keV-iMAR_ | 14,82 ± 33,87 | 11,22 ± 8,82 | 8,05 ± 10,03 | 12,03 ± 8,53 | 8,44 ± 6,83 | 17,31 ± 11,33 |
| VMI_80keV-iMAR_ | 12,75 ± 26,37 | 8,15 ± 6,11 | 6,67 ± 7,61 | 8,49 ± 6,41 | 6,67 ± 4,98 | 13,78 ± 8,93 |
| VMI_90keV-iMAR_ | 11,33 ± 21,26 | 7,02 ± 4,56 | 5,85 ± 6,65 | 6,96 ± 5,62 | 5,91 ± 4,6 | 12,12 ± 7,85 |
| VMI_100keViMAR_ | 11,08 ± 17,72 | 6,28 ± 4,66 | 5,4 ± 6,17 | 6,96 ± 5,79 | 6,09 ± 5,56 | 11,62 ± 7,34 |
| VMI_110keV-iMAR_ | 10,52 ± 15,49 | 6,09 ± 4,56 | 5,12 ± 5,82 | 6,96 ± 6,06 | 6,03 ± 5,9 | 11,29 ± 7,25 |
| VMI_120keV-iMAR_ | 10,08 ± 13,96 | 6,01 ± 4,52 | 4,94 ± 5,55 | 6,97 ± 6,27 | 5,94 ± 6,04 | 11,13 ± 7,37 |
| VMI_130keV-iMAR_ | 9,77 ± 12,84 | 5,93 ± 4,51 | 4,82 ± 5,39 | 6,99 ± 6,46 | 5,86 ± 6,1 | 11 ± 7,49 |
| VMI_140keV-iMAR_ | 9,55 ± 12,09 | 5,91 ± 4,49 | 4,76 ± 5,29 | 7,02 ± 6,64 | 5,78 ± 6,12 | 10,88 ± 7,52 |
| VMI_150keV-iMAR_ | 9,37 ± 11,49 | 5,89 ± 4,48 | 4,71 ± 5,15 | 7,06 ± 6,79 | 5,72 ± 6,14 | 10,79 ± 7,53 |
| VMI_160keV-iMAR_ | 9,22 ± 11,05 | 5,88 ± 4,48 | 4,64 ± 5,09 | 7,1 ± 6,88 | 5,66 ± 6,13 | 10,71 ± 7,54 |
| VMI_170keV-iMAR_ | 9,11 ± 10,71 | 5,85 ± 4,49 | 4,6 ± 5 | 7,09 ± 6,98 | 5,63 ± 6,13 | 10,65 ± 7,55 |
| VMI_180keV-iMAR_ | 9,03 ± 10,45 | 5,85 ± 4,48 | 4,58 ± 4,98 | 7,15 ± 7,06 | 5,59 ± 6,13 | 10,61 ± 7,55 |
| VMI_190keV-iMAR_ | 8,93 ± 10,24 | 5,83 ± 4,48 | 4,54 ± 4,94 | 7,17 ± 7,11 | 5,56 ± 6,12 | 10,57 ± 7,56 |

Suppl. Table 3b: Corrected image noise as mean± standard deviation for different ROIs in patients with spinal implants.

Supplementary Table 4

a) Qualitative artifact in hip implants

|  | Overall diagnostic image quality | Bone | Adjacent muscle and soft tissue | Bladder wall | Rectal wall | Vascular contrast |
| --- | --- | --- | --- | --- | --- | --- |
| Mixed | 3.79 [3.64 - 3.95] | 3.19 [3.04 - 3.35] | 3.91 [3.75 - 4.08] | 3.4 [3.17 - 3.63] | 2.43 [2.22 - 2.64] | 2.24 [2.12 - 2.35] |
| VMI_50keV_ | 4.46 [4.36 - 4.57] | 3.95 [3.81 - 4.09] | 4.61 [4.51 - 4.72] | 4.01 [3.83 - 4.2] | 3.03 [2.79 - 3.27] | 1.37 [1.25 - 1.5] |
| VMI_70keV_ | 4.24 [4.12 - 4.35] | 3.66 [3.52 - 3.8] | 4.42 [4.29 - 4.54] | 3.85 [3.65 - 4.05] | 2.68 [2.45 - 2.9] | 2.32 [2.19 - 2.46] |
| VMI_110keV_ | 3.94 [3.8 - 4.08] | 3.22 [3.08 - 3.37] | 3.99 [3.82 - 4.15] | 3.37 [3.14 - 3.59] | 2.41 [2.19 - 2.63] | 4.24 [4.13 - 4.35] |
| VMI_140keV_ | 3.83 [3.69 - 3.98] | 2.92 [2.76 - 3.08] | 3.76 [3.6 - 3.93] | 3.19 [2.96 - 3.41] | 2.27 [2.08 - 2.46] | 4.88 [4.82 - 4.95] |
| VMI_190keV_ | 3.83 [3.69 - 3.98] | 2.83 [2.67 - 2.99] | 3.67 [3.5 - 3.84] | 3.17 [2.95 - 3.39] | 2.24 [2.05 - 2.44] | 4.97 [4.93 - 5.02] |
| Mixed_iMAR_ | 1.6 [1.49 - 1.72] | 1.63 [1.51 - 1.74] | 1.48 [1.38 - 1.59] | 1.22 [1.14 - 1.29] | 1.08 [1.03 - 1.13] | 2.22 [2.11 - 2.32] |
| VMI_50keV-iMAR_ | 2.64 [2.49 - 2.79] | 2.24 [2.08 - 2.39] | 2.4 [2.26 - 2.55] | 1.97 [1.81 - 2.13] | 1.37 [1.25 - 1.48] | 1.36 [1.26 - 1.46] |
| VMI_70keV-iMAR_ | 2.12 [1.97 - 2.27] | 1.99 [1.83 - 2.14] | 1.95 [1.82 - 2.08] | 1.58 [1.45 - 1.72] | 1.22 [1.14 - 1.31] | 2.53 [2.4 - 2.65] |
| VMI_110keV-iMAR_ | 1.95 [1.81 - 2.09] | 1.58 [1.45 - 1.71] | 1.55 [1.43 - 1.67] | 1.29 [1.19 - 1.39] | 1.12 [1.05 - 1.19] | 4.27 [4.16 - 4.39] |
| VMI_140keV-iMAR_ | 1.92 [1.78 - 2.06] | 1.43 [1.32 - 1.54] | 1.45 [1.35 - 1.56] | 1.26 [1.17 - 1.35] | 1.1 [1.04 - 1.16] | 4.89 [4.83 - 4.95] |
| VMI_190keV-iMAR_ | 1.96 [1.81 - 2.1] | 1.37 [1.28 - 1.47] | 1.45 [1.35 - 1.56] | 1.27 [1.18 - 1.35] | 1.1 [1.04 - 1.16] | 4.96 [4.91 - 5] |
|  |  |  |  |  |  |  |
| p-values |  |  |  |  |  |  |
| VMI_190keV_ vs Mixed | 0.773 | 0.003 | 0.054 | 0.186 | 0.398 | <0.001 |
| Mixed_iMAR_ vs Mixed | <0.001 | <0.001 | <0.001 | <0.001 | <0.001 | 0.933 |
| Mixed_iMAR_ vs VMI_190keV_ | <0.001 | <0.001 | <0.001 | <0.001 | <0.001 | <0.001 |
| VMI_190keV-iMAR_ vs VMI_190keV_ | <0.001 | <0.001 | <0.001 | <0.001 | <0.001 | 0.516 |
| Mixed_iMAR_ vs VMI_190keV-iMAR_ | 0.001 | 0.001 | 0.593 | 0.494 | 0.718 | <0.001 |
| VMI_50keV-iMAR_ vs Mixed | <0.001 | <0.001 | <0.001 | <0.001 | <0.001 | <0.001 |
| VMI_50keV-iMAR_ vs VMI_190keV_ | <0.001 | <0.001 | <0.001 | <0.001 | <0.001 | <0.001 |
| VMI_50keV-iMAR_ vs VMI_190keV-iMAR_ | <0.001 | <0.001 | <0.001 | <0.001 | <0.001 | <0.001 |

Suppl. Table 4a: Subjective Evaluation results of qualitative artifact as mean with lower and upper 95%-confidence interval in patients with hip implants. Comparisons of displayed p-values were made with pairwise Mann-Whitney test with Benjamini-Hochberg adjustment.

b) Qualitative artifact in spinal implants

|  | Overall diagnostic image quality | Bone | Adjacent muscle and soft tissue | Organ margin sharpness | Prevertebral structures | Vascular contrast |
| --- | --- | --- | --- | --- | --- | --- |
| Mixed | 3.47 [3.1 - 3.84] | 3.44 [3.16 - 3.71] | 3.92 [3.61 - 4.23] | 2.29 [2.08 - 2.5] | 3.94 [3.65 - 4.23] | 2.21 [2.08 - 2.34] |
| VMI_50keV_ | 4.15 [3.9 - 4.39] | 3.98 [3.78 - 4.19] | 4.39 [4.18 - 4.59] | 2.85 [2.64 - 3.07] | 4.52 [4.32 - 4.72] | 1.66 [1.47 - 1.86] |
| VMI_70keV_ | 3.82 [3.54 - 4.1] | 3.68 [3.44 - 3.91] | 3.98 [3.69 - 4.27] | 2.47 [2.28 - 2.66] | 4.11 [3.88 - 4.35] | 3.18 [3.01 - 3.34] |
| VMI_110keV_ | 3.42 [3.15 - 3.69] | 3.19 [2.95 - 3.44] | 3.48 [3.16 - 3.81] | 2.35 [2.14 - 2.57] | 3.53 [3.23 - 3.84] | 4.45 [4.29 - 4.62] |
| VMI_140keV_ | 3.39 [3.13 - 3.64] | 2.94 [2.68 - 3.19] | 3.13 [2.79 - 3.47] | 2.26 [2.04 - 2.48] | 3.27 [2.94 - 3.61] | 4.95 [4.9 - 5.01] |
| VMI_190keV_ | 3.39 [3.13 - 3.64] | 2.84 [2.58 - 3.1] | 3 [2.67 - 3.33] | 2.27 [2.05 - 2.5] | 3.16 [2.81 - 3.51] | 5 [5 - 5] |
| Mixed_iMAR_ | 2.66 [2.44 - 2.88] | 2.47 [2.19 - 2.74] | 2.21 [1.94 - 2.48] | 2.19 [2 - 2.39] | 2.06 [1.83 - 2.3] | 2.32 [2.19 - 2.46] |
| VMI_50keV-iMAR_ | 3.34 [3.12 - 3.55] | 2.76 [2.49 - 3.03] | 2.84 [2.6 - 3.07] | 2.76 [2.53 - 2.98] | 2.45 [2.17 - 2.73] | 1.77 [1.59 - 1.96] |
| VMI_70keV-iMAR_ | 2.94 [2.75 - 3.12] | 2.48 [2.24 - 2.73] | 2.13 [1.89 - 2.37] | 2.45 [2.23 - 2.67] | 2 [1.78 - 2.22] | 3.26 [3.09 - 3.42] |
| VMI_110keV-iMAR_ | 2.74 [2.49 - 2.99] | 2.21 [1.95 - 2.47] | 1.89 [1.67 - 2.1] | 2.31 [2.08 - 2.53] | 1.9 [1.72 - 2.09] | 4.6 [4.46 - 4.74] |
| VMI_140keV-iMAR_ | 2.73 [2.48 - 2.97] | 2.08 [1.82 - 2.34] | 1.79 [1.6 - 1.98] | 2.27 [2.06 - 2.49] | 1.92 [1.74 - 2.1] | 4.98 [4.95 - 5.02] |
| VMI_190keV-iMAR_ | 2.76 [2.49 - 3.02] | 2.05 [1.8 - 2.3] | 1.76 [1.58 - 1.94] | 2.27 [2.05 - 2.5] | 1.95 [1.76 - 2.14] | 5 [5 - 5] |
|  |  |  |  |  |  |  |
| p-values |  |  |  |  |  |  |
| VMI_190keV_ vs Mixed | 0.614 | 0.005 | <0.001 | 0.95 | 0.003 | <0.001 |
| Mixed_iMAR_ vs Mixed | 0.004 | <0.001 | <0.001 | 0.767 | <0.001 | 0.227 |
| Mixed_iMAR_ vs VMI_190keV_ | <0.001 | 0.124 | 0.001 | 0.872 | <0.001 | <0.001 |
| VMI_190keV-iMAR_ vs VMI_190keV_ | 0.001 | <0.001 | <0.001 | 0.974 | <0.001 | 1.000 |
| Mixed_iMAR_ vs VMI_190keV-iMAR_ | 0.906 | 0.041 | 0.022 | 0.894 | 0.663 | <0.001 |
| VMI_50keV-iMAR_ vs Mixed | 0.511 | 0.004 | <0.001 | 0.028 | <0.001 | <0.001 |
| VMI_50keV-iMAR_ vs VMI_190keV_ | 0.735 | 0.975 | 0.426 | 0.022 | 0.005 | <0.001 |
| VMI_50keV-iMAR_ vs VMI_190keV-iMAR_ | 0.001 | <0.001 | <0.001 | 0.019 | 0.013 | <0.001 |

Suppl. Table 4b: Subjective Evaluation results of qualitative artifact as mean with lower and upper 95%-confidence interval in patients with spinal implants. Comparisons of displayed p-values were made with pairwise Mann-Whitney test with Benjamini-Hochberg adjustment.
